# Considering the Differential Impact of Three Facets of Organizational Health Climate on Employees' Well-Being

**DOI:** 10.1155/2015/407232

**Published:** 2015-08-25

**Authors:** Zandra M. Zweber, Robert A. Henning, Vicki J. Magley, Pouran Faghri

**Affiliations:** University of Connecticut, Storrs, CT 06269, USA

## Abstract

One potential way that healthy organizations can impact employee health is by promoting a climate for health within the organization. Using a definition of health climate that includes support for health from multiple levels within the organization, this study examines whether all three facets of health climate—the workgroup, supervisor, and organization—work together to contribute to employee well-being. Two samples are used in this study to examine health climate at the individual level and group level in order to provide a clearer picture of the impact of the three health climate facets. *k*-means cluster analysis was used on each sample to determine groups of individuals based on their levels of the three health climate facets. A discriminant function analysis was then run on each sample to determine if clusters differed on a function of employee well-being variables. Results provide evidence that having strength in all three of the facets is the most beneficial in terms of employee well-being at work. Findings from this study suggest that organizations must consider how health is treated within workgroups, how supervisors support employee health, and what the organization does to support employee health when promoting employee health.

## 1. Introduction

The impact that work has on employee health has been a topic of growing interest for more than 30 years [1]. Though many studies examine the role of individual factors as they relate to the relationship between work and health [2], we argue that organizational factors, such as organizational health climate, play a major role as well. This is in line with the Total Worker Health perspective, which is an integrated approach to workplace health promotion and health protection to advance worker health and well-being [3]. The purpose of the current study is to examine three facets of organizational health climate—the workgroup, supervisor and the organization—and whether these facets somehow act together as a component of employee health and well-being. Although another major component of employee health and well-being is the employee's life outside work, this is beyond the scope of the current paper where we seek to examine the role that the workplace has in employee health and well-being.

We define organizational health climate as “employee perceptions of active support from upper management, as well as supervisors and coworkers, for the physical and psychological well-being of employees” [4]. This conceptualization of organizational health climate suggests that there is a context for health that exists in organizations that is reflected by perceptions that employees have of the active support of health from coworkers, supervisors, and the organization itself. Individual perceptions of overall organizational health climate have already been found to relate to job stress, burnout, fatigue, and job satisfaction [4], and group-level perceptions of health climate have previously been found to relate to individual general health, body mass index, hypertension, burnout, and job satisfaction [5]. However, the specific components of health climate—the workgroup, supervisor, and organization—have not yet been examined, nor has the possibility that they could act together in influencing individual employee health and well-being outcomes.

The purpose of this study is to examine these three facets of organizational health climate as they appear in the workplace in various combinations in order to determine if strength in all three contributes to optimal employee well-being or if strength in one or two facets can compensate for what is lacking in the others. We begin by first considering each of the three facets and contributing theories that explain why each should be related to employee health and well-being. We then consider the importance of the three facets of health climate in combination as a means to assess a complex work system. This line of inquiry is then extended by considering the three facets of health climate at the aggregate group level to further examine the role of organizational health climate in employee health and well-being.

*Workgroup Facet of Organizational Health Climate.* The workgroup facet of organizational health climate assesses support for health from immediate coworkers. Coworkers, for many individuals, can be a major source of social support because of the relatively frequent interactions between an individual and his/her coworkers [6]. If this support regards health specifically rather than general social support, a necessary distinction when assessing organizational health climate, it can be expected that a continuous level of support from coworkers for an individual's health and well-being will have a direct beneficial impact on this employee's health and well-being.

A number of scientific studies have examined the impact of general coworker support on coping as well as on many negative workplace experiences. It is often looked at as a moderator in the relationship between negative experiences/workplace stress and negative workplace outcomes, suggesting that coworker support can act as a buffer in a number of situations [7]. Although some research has found that general coworker support does buffer the negative effects of a stressor, other research finds only that this form of social support directly affects health and well-being [8, 9]. Therefore, coworker support for health specifically might also have this direct relationship with employee health and well-being. Importantly, research studies on the effects of social support usually come from a “stressful life events” paradigm. Organizational stress is unique in that it is more chronic than event driven and therefore is considered to have a larger impact on employee health [6]. Persistence of workplace stressors suggests that social support and specifically support from coworkers might need to be consistently present to be influential rather than occurring only on an event-by-event basis.

Organizational health climate can be seen as a set of resources that are currently available to the employee and specific to employee health and well-being. This allows for Conservation of Resources (COR) theory to serve as a foundation for the reasoning behind why the workgroup facet of health climate would be related to employee health and well-being. COR posits that stress is the result of actual or threatened loss of resources or the lack of gained resources after an individual has invested their own resources [10]. Thus, an employee could greatly benefit from coworkers who regularly support his/her health by providing resources, both tangible and intangible, for improving and maintaining health and well-being. The presence of a constant stream of resources and support that benefits employee health and well-being would contribute to the development of a strong climate of support, as assessed by the workgroup facet of organizational health climate. 

*Supervisor Facet of Organizational Health Climate.* The supervisor facet of organizational health climate can also be understood to play a unique role. For example, it has been reported that supervisors can reduce the success of worksite stress and health intervention simply by expressing negative opinions about the usefulness of the program [11]. In their qualitative study, Saksvik and colleagues [11] highlighted the important barrier of management in the implementation of occupational stress and health interventions, noting that although the target consumers of these programs are the employees, middle management plays a large role in determining intervention success. The importance of middle management support comes out of its ability to control things such as the availability of employee time to participate in health programs as well as other health-promoting resources. Employees perceive and react to these actions, beliefs, and support from supervisors. Therefore, middle management is expected to play a major role in establishing an organization's health climate.

Similar to the previous research on coworker support and its relevance to the workgroup facet of health climate, general supervisor support has been widely studied but supervisor support specific to promotion of health has not. Although general supervisor support for employees may act differently compared to supervisor support for health specifically, general findings and frameworks from previous research on supervisor support can help inform the theoretical reasoning behind how the supervisor facet of organizational health climate will function and why it should be related to employee health and well-being. Because of the emotional, instrumental, informational, and social support that supervisors provide, as well as their ability to prevent certain job stressors, supervisors might have a significant impact on employee health [12, 13]. These latter types of supervisor support might also be applicable in the context of organizational health climate because supervisors can offer a similar type of support and encouragement for employee health and well-being, creating a positive climate for health in their workgroup(s).

Previous research on general supervisor support has found evidence for both the main effect and buffering effect of supervisor support on employee health [14]. For instance, Jones-Johnson and Johnson [15] found that supervisor support had a direct relationship with the psychosocial stress of employees although they had hypothesized supervisor support to act as a buffer. This was similar to findings in previous studies including one that found that supervisor support has a direct effect on reported psychological symptoms [16]. Based on this previous research, as well as the COR theory as explained in the previous section, it is expected that the presence of positive resources and support for health from one's supervisor will be directly related to employee health and well-being.

*Organization Facet of Organizational Health Climate.* As much as the supervisor and the workgroup members influence employees' perceptions of support for health in the workplace, the organization itself can also be expected to play a major role. In contrast to the more interpersonal nature of the support provided by either the supervisor or the workgroup members, how the organization supports employee health is more instrumental in nature. Instrumental support has been defined as involving behaviors that directly help the person in need [17]. This would include things such as the organization providing resources for health such as good health insurance coverage as well as setting programs and policies in place in the workplace for promoting and maintaining the health of employees. As found previously for the other two facets of organizational health climate, little to no research has been done to determine the specific type of support for employee health and well-being that would be exemplified in the construct of organizational health climate. However, numerous studies have examined the concept of perceived organizational support and its antecedents and consequences, as well as its role in the stressor-strain relationship. The theoretical frameworks used in these studies can be essential in understanding and predicting the role that the organizational facet of health climate could play.

Organizational Support Theory suggests that individuals have the tendency to assign humanlike characteristics to the organization itself, which in turn results in creating perceived organizational support [18]. This is exemplified by individuals feeling a sense of caring from their organization. In the context of organizational health climate, the perceptions of the employees that would matter are about how much the organization cares for, supports, and encourages employee health and well-being. These more specific forms of support from the organization, as long as they are in line with actual employee needs, can be perceived as a set of resources that are regularly available to the individual. Therefore, COR theory would suggest that a positive organizational facet of health climate will be directly related to employee health and well-being. 

*Importance of All Three Facets.* Although, as outlined above, we have carefully considered each of the facets individually and how they might impact employee health and well-being, the primary research question in present study is whether strength in all three facets of health climate is necessary to facilitate optimal employee well-being. Given that the three facets of health climate—workgroup, supervisor, and organization—are part of a single work system that is dynamic and constantly changing, their combined effects on health may be equally important to consider. It is therefore predicted that an organization that is lacking in one or more of the health climate facets will not provide the full benefits to employee health and well-being that organizations with strength in all three facets of health climate can offer. Employee well-being can be broadly defined to include aspects of mental health, burnout, and stress as well as other work-related well-being constructs such as work ability and workplace civility norms. Health climate is likely to impact these aspects of well-being, and therefore it is predicted that individuals who differ in terms of the three facets of health climate will differ in terms of their work-related well-being. Therefore, we hypothesize the following:

(H1) Employees who perceive a positive climate in all three facets of health climate will experience more positive health and workplace outcomes than employees who perceive one or more of these facets to be not as strong.

*Health Climate Facets at the Group Level.* When studying organizations and the people who work within them, it is necessary to examine the multiple levels that exist in an organization in order to fully understand the relationships that are occurring within it. Although the number of levels that exist may differ from organization to organization, generally speaking, organizations are not flat, meaning that some sense of organizational hierarchy does exist. Multilevel theories in organizational behavior can consist of any combination of individuals, dyads, teams, business units, corporations, and industries [19]. The importance of examining organizations from a multilevel perspective is that individual perceptions, actions, attitudes, and behaviors at the workplace do not exist in a vacuum, and to ignore the social context in which they occur can result in missing potentially important influences that exist within the work system.

To further examine whether strength in all three facets of health climate is necessary for optimal employee well-being, we also sought to examine group perceptions of health climate. Organizational climate has been defined as the shared perception that people have of their work settings that can be based on actual or inferred events as well as practices and procedures that occur in the workplace [20, 21]. When talking about shared perceptions, this suggests that analysis should be at the group level or above rather than at the individual level. This is not to say that the individual level of analysis is inappropriate; climate is often examined at the individual level as a summary perception by individuals of the work environment that is descriptive in nature, as done here and elsewhere [22, 23]. However, we believe that, to fully understand the impact of organizational health climate, we would be remiss to only examine health climate in terms of individual perceptions (aka psychological climate [24]) and argue that health climate should also be considered as* shared* perceptions among group members (organizational climate [24]).

Hence, the current study also aims to test whether work and well-being outcomes differ as a result of group-level perceptions of the health climate facets and whether strength in all three of the facets of health climate—as operationalized at the group-level—is necessary for employee optimal well-being. Specifically, the research approach is to compare groups that are similar in certain facets of health climate but differ in one or more of the remaining facets. Examining differences among these types of groups on a set of work and health-related outcomes will allow us to examine whether all three facets of health climate at the group-level are necessary for experiencing the positive effects of a healthy workplace climate and also to determine what the effect on outcomes is when one or more of the facets are lacking. Therefore, we next hypothesize the following:

(H2) Workgroups with a positive climate in all three facets of health climate will experience more positive health and workplace outcomes than workgroups that are not as strong in one or more of these facets.

## 2. Method

### 2.1. Participants

Two separate samples and datasets were used to test Hypothesis 1 and Hypothesis 2, an individual-level dataset and a group-level dataset, respectively.

Sample 1, the individual-level dataset, was collected as part of a long-term study by the Center for the Promotion of Health in the New England Workplace [25] with the Department of Corrections (DOC) in a northeast state. The overall study, entitled Health Improvement Through Employee Control (HITEC), seeks to integrate workplace health protection (occupational health and safety) with health promotion in order to improve the health and well-being of the correctional officer population. An All-Employee Survey was administered as a paper based-survey which assessed a number of constructs in addition to the ones used in this study, including ergonomics, workplace behaviors, workplace attitudes, and individual characteristics. The final sample consisted of 263 correctional officers with a mean age of 43 years and 46% worked first shift. Males made up the majority of the sample (69%), which is indicative of true gender ratios in most corrections settings. Average job tenure of the sample was 11 years and 38% of the sample had a college degree or higher. 69% of the sample self-identified as white.

Sample 2, the group-level dataset, was collected as part of an on-going multiyear study, referred to as the Civility Among Healthcare Professionals (CAHP) project. The sample consists of correctional healthcare workers primarily in medical, dental, and mental health. The central purpose of the CAHP project is to improve the social work environment by implementing a workplace incivility training program for all employees. The CAHP project involved several waves of data collection. A baseline survey combined with social network data and qualitative feedback justified the creation of workgroups based largely on facility, shift, and discipline. The current study uses data from the third wave of data collection, which consisted of an online survey that assessed many workplace attitudes, beliefs, and behaviors in addition to the variables used in this study. The final sample used in this study consisted of 171 correctional healthcare workers nested within 42 workgroups. The sample was 72% female, which is consistent with the large proportion of female healthcare workers generally. The predominant age group of the sample was age 52–60 (30% of sample), 82% of the survey respondents were Caucasian, and 76% had a college degree or higher. Average job tenure was 9 years.

### 2.2. Measures

Item response scales are a 1 (strongly disagree) to 7 (strongly agree) Likert scale unless otherwise noted. Correlation matrices, scale descriptive statistics, and alpha reliabilities can be seen in Tables 1 and 2 for Sample 1 and Sample 2, respectively.

#### 2.2.1. Health Climate

The workgroup, supervisor, and organization facets of organizational health climate were measured using the Multifaceted Organizational Health Climate Assessment (MOHCA [4]). A sample item from this scale is “my supervisor encourages participation in organizational programs that promote employee health and well-being.” The original MOHCA scale has two workgroup items, three supervisor items, and four organization items. However, one of the supervisor items was excluded due to its effect on the alpha of the scale and results of exploratory and confirmatory factor analyses which have shown that this negatively worded item did not fit well with the other two supervisor facet items. This resulted in the overall scale in each sample consisting of two items for the workgroup facet, two items for the supervisor facet, and four items for the organizational facet.

#### 2.2.2. Work-Related Well-Being

The work-related well-being variables used in the discriminant function analyses differed by sample because, as previously stated, data for both samples were collected as part of larger projects with other goals in mind, and therefore not all well-being variables were available in each sample. Additionally, in Sample 2 some work-related variables were added in addition to well-being variables so as to extend the findings from Sample 1 to apply to other work-related variables. The variables that were used on the work-related well-being discriminant functions were the following.

### 2.3. Sample 1 Only

#### 2.3.1. Civility Norms

Civility norms were measured using the 4-item Civility Norms Questionnaire Brief [26] An example item is “rude behavior is not accepted by your coworkers.”

#### 2.3.2. Work Ability

Work ability was measured using a four-item scale [27]. The response scale ranged from 0 = cannot work to 10 = work at best. An example item is “thinking about the physical demands of your job, how do you rate your current ability to meet those demands?”

#### 2.3.3. SF-12

The short form health assessment survey (SF-12 [28]) was used to assess mental health. This survey uses 12 questions and an equation to compare the health of employees to the general population of the United States. A score of 50 on the scale is comparable to the average health of the United States and a lower score indicates poorer health.

#### 2.3.4. Depression

Depression was measured using a ten-item scale [29]. The response option for this scale ranges from 1 = rarely or none of the time to 4 = all of the time (5–7 days per week). An example item from this scale is “I felt that everything I did was an effort.” Higher scores on this scale indicate higher levels of depression.

### 2.4. Both Sample 1 and Sample 2

#### 2.4.1. Burnout

Burnout was measured using 4 items from the Oldenburg Burnout Inventory [30]. An example item from this scale is “more and more often I talk about my work in a negative way.”

#### 2.4.2. Stress

Stress in general/job stresswas measured using the 6-item Stress in General/Job Stress measure [31]. The response options for this scale were 0 = no, 1.5 = ?, and 3 = yes, meaning that higher scores on this scale indicate higher levels of stress. An example item from this scale is “in general, I think my job is pressured.”

### 2.5. Sample 2 Only

#### 2.5.1. Performance

Individual self-reported job performance was measured using four items adapted from a scale by Farh and colleagues [32]. Employees were asked the stem “How do you feel your performance is viewed by the SUPERVISOR…What does your supervisor (i.e., not you) think of…” followed by an item such as “…the quality of your work?”

#### 2.5.2. Organizational Citizenship Behaviors

OCBs were measured using two items from an interpersonal OCBs scale [33]. An example item from this scale is “I pass along work-related information to others.”

#### 2.5.3. Engagement

Engagement was measured using 10 items from the Individual Work Engagement Scale [34]. An example item from this scale is “I am immersed in my work.”

### 2.6. Analytic Strategy

To test the hypotheses, a *k*-means cluster analysis was first run on each dataset to identify groups of individuals based on the strength of the three facets of health climate. This analysis empirically identifies groups of individuals (or groups of groups in the case of Sample 2) that are maximally similar within group while simultaneously being maximally dissimilar between groups. Using the clusters identified in the *k*-means analysis, a Discriminant function analysis (DFA) was then run to determine the linear combination of well-being outcomes that best discriminated among the groups. A comparison of how each of the clusters performs on the discriminant function was then used as evidence for testing Hypotheses 1 and 2.

## 3. Results

### 3.1. Individual Level

**Figure 1:**
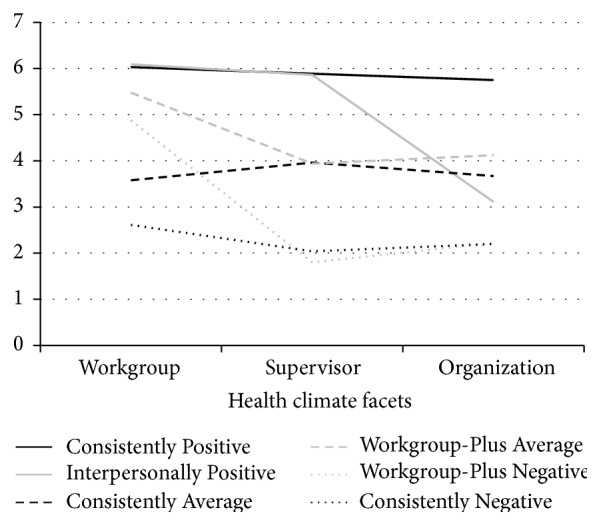
Sample 1 *k*-means cluster solution.

In the *k*-means analysis, a 6-cluster solution was retained after examining 2- through 7-cluster solutions. A 6-cluster solution was determined based on adequate cluster size and maximizing meaningful differences between clusters; see Table 3 for cluster sizes. As shown in Figure 1, there were three pairs of clusters that emerged, two clusters that were marked as mostly positive, two that were marked as mostly on average, and two that were marked as mostly negative. In the pair of mostly positive, one cluster of employees (entitled “Consistently Positive”) reported high levels of all three of the health climate facets, whereas another cluster of employees (“Interpersonally Positive”) reported high levels of both the workgroup and supervisor facets yet a lower organization facet. In the pair of average clusters, the “Consistently Average” cluster has average levels of all three of the facets while the “Workgroup-Plus Average” cluster is on average in the supervisor and organization facets yet higher in the workgroup facet. Lastly, in the negative pair of clusters, the “Consistently Negative” cluster reported low levels of all three of the health climate facets and the “Workgroup-Plus Negative” cluster is relatively high in the workgroup facet but low in the supervisor and organization facets.

Cluster membership was saved as a variable and then used as a grouping variable in a discriminant function analysis (DFA). The purpose of using DFA in the current study was to test how well linear combinations of well-being variables also differentiate between the clusters. Seven variables related to employee well-being were entered into the discriminant function analysis: SF-12 mental, job stress, depression, disengagement (burnout), exhaustion (burnout), civility norms, and work ability.

**Figure 2:**
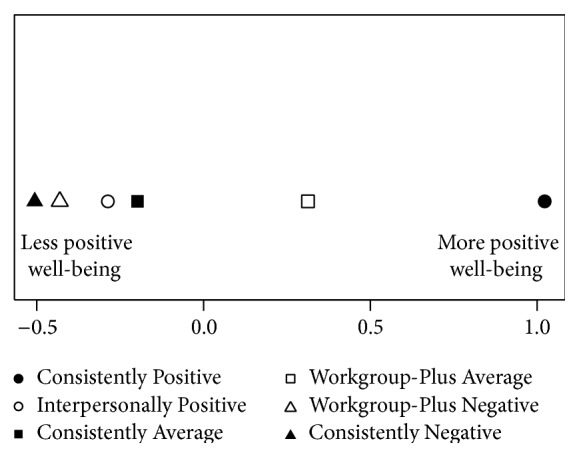
Sample 1 DFA group centroids.

One discriminant function was significant (Wilks' lambda = 0.685, *p* < 0.001) and accounted for 64% of the variance among the groups. This function was defined with a positive correlation with civility norms (*r* = 0.89), a positive correlation with work ability (*r* = 0.39), a positive correlation with SF-12 mental (*r* = 0.34) and negative correlations with job stress (*r* = −0.34), exhaustion (−0.46), disengagement (*r* = −0.57), and depression (*r* = −0.43). This pattern of correlations indicates that more positive scores on the function are associated with more positive work-related well-being. Group centroids are plotted in Figure 2. Comparisons of how clusters perform on the function within pairs as well as comparisons between pairs in terms of outcomes can serve to address the research question of whether all three facets of health climate are necessary for optimal well-being.

Results from this analysis indicate that Hypothesis 1 was supported. Figure 2 shows that “Consistently Positive” (the cluster that was positive in all three of the facets) is the most positive of all the clusters on the function. A comparison of “Consistently Positive” to “Interpersonally Positive” shows that “Consistently Positive” performs better on the function despite the two clusters being similarly high in the workgroup and supervisor facets. Similarly, “Consistently Negative” (the cluster that was negative in all three of the facets) is the most negative of all of the clusters on the function. A comparison between “Consistently Negative” and “Workgroup-Plus Negative” shows that these two clusters were quite similar on the discriminant function. A comparison of “Workgroup-Plus Average” and “Consistently Average” indicates that “Workgroup-Plus Average” is more positive on the function than “Consistently Average.” In addition to the comparisons within the three pairs, a comparison of “Workgroup-Plus Negative” and “Interpersonally Positive” indicates that “Interpersonally Positive” is more positive on the function than “Workgroup-Plus Negative,” which highlights the importance of supervisory support of employee health with respect to overall employee well-being. Most puzzling is that Figure 2 also shows that “Consistently Average” and “Interpersonally Positive” are similar on the function despite their differences on the workgroup and supervisor facets. This last result is looked at closely in Section 5.

### 3.2. Group Level

Hypothesis 2 posits that workgroups with a positive climate in all three facets of health climate will experience more positive health and workplace outcomes than workgroups who are not as strong in one or more of these facets. A 6-cluster solution was initially examined, as this was the number of clusters determined when examining Hypothesis 1. However, due to a much lower sample size in Sample 2 (as sample size was determined by the number of groups, *n* = 42), the 6-cluster solution did not yield meaningful results. A 4-cluster solution was retained after examining 2- through 5-cluster solutions yielding similar clusters—a positive cluster, a negative cluster, and two relatively average clusters.

**Figure 3:**
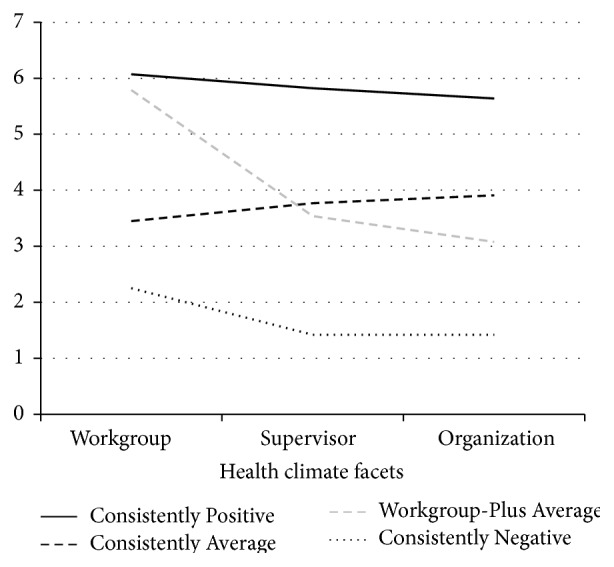
Sample 2 final clusters.

The 4-cluster solution is shown in Figure 3. Again, one cluster of employees (“Consistently Positive”) reported high levels of all three of the health climate facets and one cluster of employees (“Consistently Negative”) reported low levels of all three of the health climate facets. Employees clustering in “Consistently Average” and “Workgroup-Plus Average” groups reported similar levels of the supervisor and organization facet of health climate but “Workgroup-Plus Average” reported higher levels of workgroup health climate than “Consistently Average.” Cluster sizes are shown in Table 3.

Cluster membership was saved as a variable and then used in the full dataset, which included individual and group data, as a grouping variable in a discriminant function analysis. Individual-level outcomes were entered into this discriminant function analysis to test whether health climate facet clusters affected employee well-being and work-related outcomes. The five outcome variables that were entered into the discriminant function analysis were burnout, stress, performance, engagement, and organizational citizenship behaviors.

One discriminant function was significant (Wilks' lambda = 0.80, *p* < 0.01) and accounted for 67.4% of the variance among the clusters. This function was defined with a positive correlation with employee performance (*r* = 0.70), a positive correlation with citizenship behaviors (*r* = 0.38), and a positive correlation with individual engagement (*r* = 0.51). This function was also defined with negative correlations with job stress (*r* = −0.80) and burnout (*r* = −0.49). This pattern of correlations indicates that more positive scores on the function are associated with more positive well-being and work-related outcomes.

**Figure 4:**
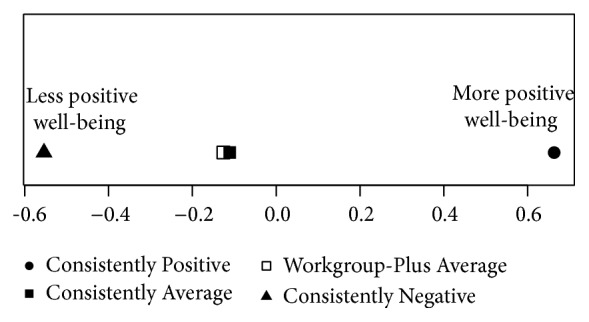
Sample 2 discriminant function analysis group centroids.

Group centroids on this significant function are plotted in Figure 4. Results from this analysis indicate that Hypothesis 2 was supported. Figure 4 shows that “Consistently Positive” fell at the most positive end of this function. Similarly, “Consistently Negative” fell at the most negative end of this function. Interestingly, “Consistently Average” and “Average-Higher Workgroup” do not significantly differ on this function, even though “Average-Higher Workgroup” has higher levels of the workgroup facet. However, “Consistently Positive” has a significantly more positive score on the function than “Average-Higher Workgroup” even though these two clusters have similar scores on the workgroup facet but differ in that “Average-Higher Workgroup” has lower scores on the supervisor and organization facets. These results suggest that having high scores in each of the three facets is most beneficial for the outcomes of performance, engagement, organizational citizenship behaviors, burnout, and job stress.

## 4. Discussion

The purpose of this study was to determine whether all three facets of health climate—the workgroup, supervisor, and organization—work together to contribute to employee well-being. Although this study does not focus on the many influences on individual health and well-being outside of a work organization, some interesting findings emerged that can guide organizations which are interested in promoting the health and well-being of its workers.

Findings from the *k*-means cluster analysis at the individual worker level suggest that health climate indeed differs for groups of employees and that these groups also vary substantively on organizationally relevant outcomes. Specifically, emergent employee groups noticeably fell into three pairs: (1) those Consistently Positive or at least Interpersonally Positive, (2) those Consistently Average or on average those with greater support from workgroup members, or (3) those Consistently Negative, or negative with greater support from workgroup members. For both Sample 1 and Sample 2, results of the DFA indicate that the cluster that was high in all three of the MOHCA facets had a much higher score on the overall health and well-being function than the other clusters and the cluster that was low in all of the three MOHCA facets had the lowest score on the overall health and well-being function. These duplicate findings at both the individual and group levels highlight the importance of having strength in all three of the MOHCA facets for employee health and well-being.

In comparing the remaining clusters in the individual-level sample, some interesting findings emerged from the breakdown of the pairs of employee groups. First in terms of the positive pair, the results provide clear evidence that strength in all three of the facets is most beneficial to both organizations and employees. A comparison between the two clusters in this pair, “Consistently Positive” and “Interpersonally Positive,” shows that great strength in two facets alone cannot fully compensate for weakness in the remaining facet. The “Interpersonally Positive” cluster differed from “Consistently Positive” only in that it had a lower score on the organization facet; yet the “Interpersonally Positive” cluster was much more negative on the DFA function than was the “Consistently Positive” cluster. This result suggests that employees' well-being suffers considerably when organizational support of their health is lacking, even in the presence of both strong supervisory support and strong coworker support of health.

A comparison of the average pair of clusters, “Consistently Average” and “Workgroup-Plus Average” to each other as well as a to the “Consistently Positive” cluster, bolsters the importance of organizations needing to strive to have more than a mediocre health climate. The “Consistently Average” and “Workgroup-Plus Average” clusters fell towards the center of the DFA function, much lower than the “Consistently Positive” cluster. Also the “Workgroup-Plus Average” cluster was more positive on the DFA function than the “Consistently Average” cluster. These findings suggest that strength in one of the facets alone cannot fully compensate for mediocrity in the other two facets; however, strength in one facet can result in slightly better health and well-being outcomes. Findings from all of these comparisons of the average clusters support the notion that having strength in all three of the health climate facets is most beneficial and that organizations could greatly benefit from taking steps to improve facets of health climate that only receive average scores on the MOHCA.

Not surprisingly, a comparison of the negative pair, “Consistently Negative” and “Workgroup-Plus Negative,” corroborates the above findings. These two clusters performed the worst on the DFA function in comparison to the pair of average clusters and the pair of positive clusters. Interestingly, “Consistently Negative” and “Workgroup-Plus Negative” lie close to each other on the function, suggesting that when an organization has low scores on one or more of these MOHCA facets, this may overshadow any benefits from average or higher scores on one or more of the remaining facets. This is a key finding, not only because it supports our earlier finding that strength in one cluster cannot compensate for weakness in another cluster, but also because it indicates that a low score on one or more of the MOHCA facets is detrimental and therefore should be prioritized when planning interventions to improve organizational health climate.

Findings from the group-level *k*-means cluster analysis in Sample 2 are very similar to the findings from Sample 1, demonstrating that workgroups can also differ among the facets of health climate, for example, being high in the workgroup facet yet lower in the supervisor and organization facets. A four-cluster solution was extracted in the analyses used to test Hypothesis 2. Although the number of workgroups in each cluster was lower than conventional standards for *k*-means cluster analysis, because this analysis accounted for the number of people nested within these groups, this is not thought to be a limiting factor in this study.

Similar to the detailed individual-level results, at the group-level the “Consistently Positive” cluster that was high in all three of the MOHCA facets was positioned most positively on the health and well-being function, and the “Consistently Negative” cluster which was low in all three facets was positioned most negatively on the function. Interestingly, “Consistently Average” and “Workgroup-Plus Average” did not differ on the function. This was initially an unexpected result because “Workgroup-Plus Average” had significantly higher scores on the workgroup facet than “Consistently Average.” However, this finding is still consistent with the findings from Sample 1 in that strength in one MOHCA facet was unable to compensate for weakness in one or more of the other MOHCA facets. Similarly, “Workgroup-Plus Average” can be compared to the “Consistently Positive” cluster which was high in all three facets, because these two clusters have similarly high levels of the workgroup facet but they differ in that “Workgroup-Plus Average” is significantly lower on the supervisor and organization facets. Results from the discriminant function analysis show that “Consistently Positive” has a more positive score on the health and well-being function than “Workgroup-Plus Average.” The interpretation of this comparison result in combination with the interpretation of the comparison between “Consistently Average” and “Workgroup-Plus Average” suggests that strength in all three MOHCA facets is an important determinant of outcomes regarding job stress, burnout, engagement, performance, and organizational citizenship behaviors.

Together, findings from the Sample 1 and Sample 2 *k*-means analysis and DFA point to the importance of strength in all three facets of health climate at the same time. Findings from these two different samples suggest that strength in all three of the facets leads to more favorable outcomes than when one or more of the MOHCA facets are not strong. This is an important finding for organizations and researchers because, for example, just because an organization has the resources for an employee health program and sponsors many health-related events, this is not likely to be very effective if its supervisors do not support employee health. Therefore, organizations cannot solely rely on top-down efforts to cultivate a healthy workplace climate. Instead, support for health will also need to come from all levels of the organization.

One alternative approach to adopting top-down interventions to benefit employee health is to support a grassroots intervention planning approach that involves employees self-identifying shared health concerns as well developing intervention ideas that could improve coworker support for health consistent with Total Worker Health [35]. Interventions coming out of this participatory design process are intended to be integrated by involving changes in the workplace and work organization as well as changes in employee behavior. This same approach for designing integrated interventions could be used to address a lack of supervisor support of workplace health.

Additionally, the finding that strength in one MOHCA facet cannot compensate for lacking strength in one or more of the other MOHCA facets suggests a unique contribution of each of these three facets and that all three facets are integral to health climate. A workgroup that is supportive for health cannot alone compensate for an organization that does not provide the resources for its employees to be healthy and supervisors who do not support their employees' health. This finding is important because it suggests the importance of first considering each of the three MOHCA facets separately and then determining how to gain strength in each. Similarly, the finding that a low score in any of the three facets leads to the cluster being much lower on the DFA health and well-being function suggests the need for organizations to identify where their primary weaknesses might be in health climate and to then prioritize interventions to address these weaknesses accordingly.

From a Conservation of Resources theory perspective, an organization must have some level of each of the three facets to support employee needs. Weakness in one or more of the three MOHCA facets suggests an inadequate amount of resources in that facet to support employee health subsequently leading to the possibility of less optimal employee well-being. On the other hand, strength in all three MOHCA facets can provide continuous resources for employee health from multiple levels within the organization in order to proactively support employee health and well-being. This level of support for health from workgroup members, supervisors, and the organization is something able to be perceived by employees and therefore can have an impact not only on health and well-being but also on work-related attitudes.

Overall, the results of the present study suggest that assessment of organizational health climate is important and that additional consideration of each of the three MOHCA facets is useful for achieving a healthy organization. After assessing health climate and determining whether the three facets are positive, on average, or low should determine the action steps for organizational improvement. For example, if an organization finds that their workgroup facet of MOHCA is weak but the supervisor and organization facets are both on average, this organization would benefit most from supporting an intervention designed specifically to target coworker support for health. However if an organization assesses their health climate and finds that all of the MOHCA facets are on average, this organization might benefit from a more generalized intervention that targets coworkers, supervisors, and the organization.

## 5. Limitations and Future Research

Although the strength of the current study rests in its exploration of two samples, one at the individual level and one at the group level, both consisted of cross-sectional data, and participants in both samples worked in a similar environment. Future research examining the three facets of health climate over time could help determine how strength in these three facets unfolds and whether the MOHCA facets can influence each other over time and how they are related to other well-being related variables such as work sense of coherence [36]. Replicating and extending the findings from the current study to other work samples would also strengthen the case that organizations need to focus on more than one facet of health climate. Additionally, now that there is evidence that all three facets are important in combination, future research studies could examine how best to develop interventions to target specific areas of health climate that are lacking. New intervention approaches could also be developed that benefit all three facets of health climate in a more comprehensive manner.

## 6. Conclusion

The overall combined results from analyses performed on Sample 1 and Sample 2 in the present study have highlighted the importance of considering all levels of the work system when thinking about the context for health that occurs in an organization. Understanding how the three facets of health climate can work together provides an important piece to the puzzle of what organizational health climate consists of and also why organizations should care about it. With increasing attention on health in today's workplace, further research using MOHCA to assess organizational health climate could help contribute to a better understanding of this phenomenon as well as further translate these research findings into practice through targeted interventions that create healthier organizations and employees.

## Figures and Tables

**Table 1:** Sample 1 correlation table.

	M	SD	1	2	3	4	5	6	7	8	9
(1) Workgroup HC	4.59	1.46	(0.57)								
(2) Supervisor HC	3.61	1.63	0.42^*∗∗*^	(0.93)							
(3) Organization HC	3.59	1.41	0.44^*∗∗*^	0.71^*∗∗*^	(0.88)						
(4) Civility norms	4.05	1.3	0.33^*∗∗*^	0.34^*∗∗*^	0.43^*∗∗*^	(0.86)					
(5) Work ability	8.79	1.3	0.20^*∗∗*^	0.03	0.16^*∗∗*^	0.20^*∗∗*^	(0.90)				
(6) Job stress	1.2	0.81	−0.13^*∗*^	−0.09	−0.13^*∗*^	−0.09	−0.31^*∗∗*^	(0.84)			
(7) Depression	1.52	0.47	−0.19^*∗∗*^	−0.19^*∗∗*^	−0.25^*∗∗*^	−0.23^*∗∗*^	−0.51^*∗∗*^	0.29^*∗∗*^	(0.77)		
(8) SF-12 Mental	48.77	10.89	0.16^*∗∗*^	0.07	0.16^*∗∗*^	0.23^*∗∗*^	0.50^*∗∗*^	−0.34^*∗∗*^	−0.68^*∗∗*^	NA	
(9) Burnout	3.98	1.25	−0.15^*∗*^	−0.20^*∗∗*^	−0.25^*∗∗*^	−0.34^*∗∗*^	−0.37^*∗∗*^	0.39^*∗∗*^	0.51^*∗∗*^	−0.54^*∗∗*^	−0.8

Note: *∗* indicates *p* < 0.05 and *∗∗* indicates *p* < 0.01.

**Table 2:** Sample 2 correlation table.

	M	SD	1	2	3	4	5	6	7	8
*Grouplevel *										
(1) Workgroup facet	4.73	0.81								
(2) Supervisor facet	3.74	0.84	0.60							
(3) Organization facet	3.63	0.83	0.58	0.85						
*Individual-level outcomes *										
(4) Burnout	4.59	1.33	−0.19	−0.17	−0.21	(0.71)				
(5) Job stress	1.83	0.90	−0.17	−0.26	−0.33	0.40	(0.81)			
(6) Performance	3.93	0.68	0.27	0.19	0.26	−0.15	−0.22	(0.88)		
(7) Engagement	5.10	1.28	0.20	0.21	0.22	−0.67	−0.36	0.23	(0.93)	
(8) OCB-E	6.10	0.96	0.19	0.13	0.17	−0.33	−0.13	0.30	0.46	(0.85)

Note: *p* values are not reported because of the aggregate variables. Coefficient alpha presented on the diagonal for the individual-level variables.

**Table 3:** Number of individuals (Sample 1) or groups (Sample 2) per cluster.

Cluster	Sample 1	Sample 2
Consistently Positive	36	12
Interpersonally Positive	11	
Consistently Average	62	7
Workgroup-Plus Average	64	15
Consistently Negative	41	8
Workgroup-Plus Negative	49	
